# Concentration of Selected Essential and Toxic Trace Elements in Horse Hair as an Important Tool for the Monitoring of Animal Exposure and Health

**DOI:** 10.3390/ani12192665

**Published:** 2022-10-04

**Authors:** Dorota Cygan-Szczegielniak, Karolina Stasiak

**Affiliations:** Department of Animal Physiology and Physiotherapy, Faculty of Animal Breeding and Biology, Bydgoszcz University of Science and Technology, Mazowiecka 28, 85-084 Bydgoszcz, Poland

**Keywords:** trace elements, toxic elements, horses, hair, oat

## Abstract

**Simple Summary:**

Heavy metals pose a serious risk to the normal functioning of living organisms. Long-term exposure to heavy metals may lead to many pathologies and various dysfunctions. On the other hand, heavy metals are components of many enzymes, so any anomalies in their concentrations are not desirable. The main purpose of the study was to analyse the concentrations of selected essential and toxic trace elements in the hair of sports and recreational riding horses from studs located in central Poland. We also measured the concentration of heavy metals in oats used as the basic component of the equine diet to investigate the interactions between the elements in the feed and hair. Assessment of essential heavy metals is important for the monitoring of exposure and the health of horses. The study confirmed many significant relationships between the concentrations of elements in the hair and oats, as well as interactions between the content of these xenobiotics in feed and the analytical matrix.

**Abstract:**

The main purpose of the study was to analyse the concentrations of selected essential and toxic trace elements in the hair of sports and recreational riding horses from studs located in central Poland and thus test the usefulness of this matrix for monitoring the exposure of these animals. We also measured the concentration of heavy metals in oats used as a basic component of the equine diet to investigate the interactions between these elements in the feed and hair. The basic chemical composition of oats was analysed. Elemental analysis was performed using an EcaFlow 150 GLP electrochemical analyser with an E-104L electrode and reference to a calibration curve. The chemical composition of oats was investigated by near infrared transmission (NIR) spectroscopy calibrated for an artificial neural network (ANN) using a Foss InfraXact spectrometer. Among all elements, the coefficient of variation was highest for Pb and lowest for Cd, regardless of the study group. The content of elements in horse hair was in the range of 153.56 to 185.79 mg·kg^−1^ for Zn, 6.10 to 11.99 mg·kg^−1^ for Cu, 0.578 to 0.813 mg·kg^−1^ for Pb and 0.011 to 0.015 mg·kg^−1^ for Cd (in kg of d.w.). For hair, we found a significant negative correlation for Zn-Cu (r_xy_ = −0.539) and Cd-Cu (r_xy_ = −0.676) at *p* ≤ 0.05. For feed, there was a highly significant positive correlation for Cu-Pb (r_xy_ = 0.723) and Zn-Cd (r_xy_ = 0.714) at *p* ≤ 0.01. We found significant negative oats–hair interactions for Cu-Zn, Pb-Cu, Cd-Cu and Zn-Pb, and a positive oats–hair interaction for Cu-Cu.

## 1. Introduction

Heavy metals are elements characterized by high atomic weight and high densities exceeding 5 g/cm^3^ [1]. In our study, we considered four main heavy metals with densities increasing in the order Zn, Cd, Cu and Pb. Heavy metals accumulate in different organs and tissues. They can be detected, for example, in hair, internal organs and muscles [2,3]. Toxic metals such as cadmium or lead pose a serious risk to the proper function of living organisms. These elements can have a strong negative biological effect and are classified as major environmental pollutants [4,5]. Cadmium and lead are not biodegradable, and when highly concentrated in the air, soil or water, they migrate to plants and the entire food chain [4]. Long-term exposure to these toxic elements may cause many pathologies and various dysfunctions [6,7,8]. Lead causes oxidative stress and reduces the antioxidant capacity of cells by contributing to increased production of free radicals [6]. Cadmium may have hepatotoxic, nephrotoxic and haemotoxic effects, and may promote bone degeneration and blood damage [8].

On the other hand, essential heavy metals, including copper and zinc, are involved in various biological processes and numerous enzymatic reactions, so their optimal concentrations are of key importance for the proper function of living organisms [6,7,8]. Zinc is an essential element in various physiological processes, such as DNA synthesis. It can act as an antioxidant and also influences the function of many enzymes, DNA, proteins and transcription factors [9]. Copper is primarily involved in the formation of haemoglobin, as well as the proper function of many enzymes, often as a structural component, such as, among others, superoxide dismutase, tyrosinase or cytochrome c oxidase [2,6,8]. Any anomalies in the concentration—both deficiency and excess—of copper and zinc are undesirable and may produce a toxic effect by disrupting the function or structure of many enzymes and thus tissues or organs [6,7,8]. All heavy metals enter the body mainly through the respiratory tract and the alimentary tract [2].

Hair from a horse’s mane can be used as a bioindicator of exposure to heavy metals (in particular, lead) that might not be detected in the blood. Thus, hair is a more stable analytical matrix, providing a lot of information about a given type of exposure over the years [10,11,12,13,14]. The main advantage of this biological material is its availability, which enables easy, stress-free and non-invasive sampling [2,3,14,15]. A high concentration of toxic elements in the bodies of livestock may reduce their productivity, performance and metabolism [15,16]. Therefore, the monitoring of these elements is an important tool for the assessment of animal exposure and health. Monitoring of heavy metal concentrations in tissues, organs or hair clearly demonstrates the whole-body status of a given animal and existing exposure.

The main purpose of the study was to analyse the concentrations of selected essential and toxic trace elements in the hair of sports and recreational riding horses from studs located in central Poland and thus test the usefulness of this matrix for monitoring the exposure of these animals. We also measured the concentration of heavy metals in oats used as a basic component of the equine diet. Correlations between the concentrations of heavy metals in hair and oats, as well as interactions between the content of these xenobiotics in the feed and their concentration in the analytical matrix, were also investigated.

## 2. Materials and Methods

### 2.1. Study Material

#### 2.1.1. Study Groups

Horses originated from four stables located in the Kuyavian-Pomeranian province, central Poland. It is a moderately industrialized region, and most of the largest local businesses operate in the chemical, electrical, machinery, food processing and printing sectors. The average annual concentration of lead and cadmium in the atmospheric air (0.0196 and 0.00052 µg/m^3^) did not exceed the acceptable concentrations for these metals [17].

The horses used in the experiment were kept in stalls of approx. 12 m^2^ surface area, with bedding on the floor. The stalls were located inside a stable with a door opening onto the aisle way. The cubature of each stall was about 40 m^3^. The age of analysed horses was 6–9 years. The animals had regular veterinary checks, were healthy, in good shape and had no symptoms of disease. A total of 36 horses took part in the experiment, including 13 geldings and 23 mares. Animals from research groups I, III and IV weighed (mean ± SD): 587.14 ± 24.97 kg, 590.21 ± 29.27 kg and 602.72 ± 14.89 kg, respectively. Ponies from group II weighed 201.11 ± 23.15 kg. Due to the lack of statistically significant differences in the concentration of elements between male and female horses in all studied groups, study animals were not divided into subgroups by sex. The analysed groups differed in terms of the concentration of trace elements in oats and the way in which the horses were used. For the protection of private owners’ data, the stables from which the horses came were coded as I, II, III and IV.

Horses kept in stable I (*n* = 8) were used for recreational riding, on average 3 times/week for 2–3 h/day. They were kept on straw bedding, fed three times a day with hay and oats, and occasionally fed with carrots and apples, regularly going out into the paddocks with unlimited access to drinking water. In more detail to the above information, horses in this group received around 12 kg good quality hay/day (3 × ration of 4 kg) and whole grain oats at a rate of 4 kg/day (3 × ration of 1.3 kg), carrots or apples 1 × day in the amount of about 3 kg or mixed, in similar proportions, i.e., 1:1 kg, or 1.5:1.5 kg, and electrolytes during the summer.

Horses (ponies) kept in stable II (*n* = 9) were used for recreational riding and hippotherapy, on average 3 times/week for 2–3 h/day. They were kept on straw bedding, fed twice a day with hay and oats, occasionally fed with carrots and apples, and had unlimited access to drinking water. As the ponies had access to pasture most of the day, hay was limited to 4 kg (2 × ration of 2 kg), and whole oat grain was fed 1.5 kg/day (2 × ration of 0.75 kg). In addition, the ponies were given about 2 kg of apples/carrots/day.

Horses kept in stable III (*n* = 9) were used for recreation 4–5 times/week for 1–2 h a day for the training of young riders. They were kept on straw bedding, had access to paddocks and unlimited access to drinking water, were fed three times a day with hay and oats, and were occasionally fed with carrots and apples. The average working time of the horses was not significantly different from the first group (they walked more often but within shorter time limits), so the feeding of both groups was the same. The horses received approx. 12 kg of good quality hay/day (3 × ration of 4 kg), whole grain oats at a rate of 4 kg/day (3 × ration of 1.3 kg), carrots or apples 1 × day in the amount of about 3 kg (or mixed, in similar proportions, i.e., 1:1 kg, or 1.5:1.5 kg) and electrolytes during the summer. The fact that the horses worked under a young rider does not affect the diet.

Horses kept in stable IV (*n* = 10) were used in sports and training 5–6 times/week during the competition season for no longer than 2 h/day. They were kept on sawdust bedding, had access to paddocks and unlimited access to water, were fed 3 times/day with hay and oats as well as supplements for sports horses (high-energy muesli) and vitamins. Horses in this group were provided with roughage in the form of hay, chaff, etc. In total, they received 10 kg of good quality hay (3 × ration of 3.3 kg) or 8 kg with the addition of chopped alfalfa (3 × ration of 2.6 kg), crushed oats—approx. 4 kg/day (3 × ration of 1.3 kg)—with the addition of muesli, according to the manufacturer’s recommendation, usually 1 kg/day (the whole ration of muesli was served in the morning). They were also fed vitamins and other supplements as required (magnesium, electrolytes in summer, biotin, MSM, etc.).

All horses had quarterly biochemical blood tests to monitor their health.

#### 2.1.2. Hair Samples

The analytical material was horse hair samples (*n* = 36) collected in the spring during intense moulting (March-April). Hair was taken from an area of 15 × 15 cm just behind the *arcus costalis*, preserved for further analysis in sealed polyethylene bags and stored in a dry and shaded place. Hair samples were collected from the animals during combing, which reduced the stress associated with the sampling procedure. The hair was cut close to the skin from the horse’s mane using special stainless-steel scissors. This procedure ensured sampling without the risk of chemical contamination, which is important in toxicological studies.

#### 2.1.3. Oats Samples

Due to the fact that oats were the main component of the diet of all tested horses and the basic component of feed rations, the concentration of essential and toxic trace elements was also determined in this matrix. The oats were obtained directly from the stable owners, and it was the same product that was normally fed to the horses.

### 2.2. Sample Preparation

To remove dirt and grease from the hair, the samples were washed with acetone and placed in an ultrasonic bath for 15 min, removed and stored for 12 h. Acetone was removed by decantation. The hair was rinsed twice with distilled water and dried in an oven at a temperature below 50 °C. An appropriate procedure for preparing the hair for analysis ensured that samples were free from chemical contamination. The hair and oat samples were wet-digested using a microwave digestion system (EthosPlus, Milestone, Sorisole, Italy), with the option of ATC-300 automatic temperature control, according to Polish Standard PN-EN 13805:2014 [18]. For this purpose, 0.20 g hair samples and 1 g ground oat samples were prepared, then treated with 6.25 cm^3^ of a mixture of 65% HNO_3_ and 30% H_2_O_2_ in a 4:1 volume ratio (*v*:*v*). Samples were digested for 20 min. The temperature was slowly increased to 190 °C in the first 10 min and then maintained at 190 ± 5 °C. After digestion, samples were completely transferred to 25 mL volumetric flasks and the volume was adjusted with distilled water. Then, 25 mL of the prepared digest was poured into 50 mL flasks, and 0.4 mL of HCl was added and adjusted up to the mark with deionized water. A blank sample was prepared by mixing 0.4 mL of HCl and deionized water in the same proportions, as in the sample preparation procedure.

Elemental analysis was performed using an EcaFlow 150 GLP electrochemical analyser (Istran, Slovakia) with an E-104L electrode (working with an EcaCell 104) and reference to a calibration curve. For Zn, Cu, Cd and Pb measurements, the solutions for calibration were prepared using certified standard solutions from Merck KGaA, i.e., Zn reference solution Zn(NO_3_)_2_ in HNO_3_ 0.5 mol·L^−1^ in concentrations: 25, 50, 100 and 200 µg·L^−1^; Cu(NO_3_)_2_ in HNO_3_ 0,5 mol·L^−1^ in concentrations: 2.5, 5.0, 10.0 and 20.0 µg·L^−1^; Cd(NO_3_)_2_ 0.5 mol·L^−1^ in concentrations: 0.5, 2.5, 5.0 and 10.0 µg·L^−1^, and Pb(NO_3_)_2_ in HNO_3_ 0.5 mol·L^−1^ in concentrations: 2.5, 5.0, 10.0 and 20.0 µg·L^−1^. R-013 electrolyte was used to prepare reference standards. Laboratory control procedures for the measurements were performed for each series. All concentrations of heavy metals in the analysed matrices were higher than LOD. The content of Zn, Cu, Pb and Cd in hair samples was expressed in mg·kg^−1^ of dry weight (d.w.).

The chemical composition of oats was analysed by near infrared transmission (NIR) spectroscopy calibrated for an artificial neural network (ANN) using the Foss InfraXact spectrometer (Foss, Germany). Measurements were carried out in the Laboratory of Chemical Research and Instrumental Analyses of the Bydgoszcz University of Science and Technology.

### 2.3. Statistical Analysis

Most of the data did not meet the requirements of normal distribution, as verified by the Shapiro–Wilk test, or the requirements of homogeneity of variance measured with Levene’s test necessary for parametric tests. Therefore, in order to investigate the significance of differences between multiple independent samples (groups), we used non-parametric analysis (non-parametric ANOVA), the Kruskal–Wallis test and the median test, as well as multiple comparisons of mean ranks for all samples. Correlations between selected parameters were analysed using Spearman’s rank correlation coefficients (in the hair, oat and hair vs. oat). The coefficient of variation (CV) was also calculated to illustrate the variation of the analysed trace elements in individual samples. The obtained data were processed using Statistica 13.1 software (StatSoft, Krakow, Poland).

## 3. Results

Table 1 presents the content of selected essential and toxic trace elements in the hair of horses from four stables located in the same region of Poland. Groups I and III comprised recreational riding horses, group II were ponies and group IV were sports horses. The content of Zn in the horse hair was in the range of 153.56 to 185.79 mg·kg^−1^ of d.w., and the highest concentrations were measured in sports horses. Significant differences in Zn concentrations were found only between ponies and sports horses (*p* ≤ 0.05). The content of Cu was in the range of 6.10 to 11.99 mg·kg^−1^ of d.w., and the highest concentrations were measured in the hair of recreational riding horses from group I. There were significant differences (*p* ≤ 0.01) in Cu concentrations in hair between recreational riding horses from group I, ponies from group II and recreational riding horses from group III. We also found very significant differences in the content of Cu in hair between ponies and sports horses (*p* ≤ 0.01). The content of Pb was in the range of 0.578 to 0.813 mg·kg^−1^ of d.w. and did not differ significantly between groups. Considering all analysed trace elements, the coefficient of variation was highest for Cu (Table 1). The concentration of Cd in the hair of horses was the lowest and ranged from 0.011 to 0.015 mg·kg^−1^ of d.w. Hair analysis revealed significant differences between the recreational riding horses, ponies and sports horses (*p* ≤ 0.05). The coefficient of variation was lowest for Cd.

In order to verify the relationship between toxic and essential trace elements in the hair of horses, Table 2 presents correlation coefficients regardless of the study group. A significant negative correlation was found only for Zn-Cu and Cu-Cd (r_xy_ = −0.539 and r_xy_ = −0.676, respectively; *p*≤ 0.05).

Table 3 presents the concentrations of selected essential and toxic trace elements in the equine feed sampled from the same stables where horses were kept. Oats were the major component of the equine diet, and for this reason they were analysed for the concentration of the same trace elements as in the case of hair samples. The content of Zn in the oats fed to horses was in the range of 58.06 do 87.84 mg·kg^−1^ of d.w. Despite the large scatter of values measured for Zn, statistical analysis did not reveal any significant differences between the groups. The content of Cu ranged from 11.85 to 19.36 mg·kg^−1^ of d.w. and was highest in the oats fed to horses from group I. There were very significant differences (*p* ≤ 0.01) in Cu concentrations between group II and other groups. Similarly to hair analysis, no significant differences in the concentrations of Pb were found between groups, and the values ranged from 0.495 to 0.636 mg·kg^−1^ of d.w. This toxic metal, as in the case of hair analysis, was characterized by the highest coefficient of variation among all investigated trace elements (Table 3). The content of Cd in horse feed was in the range of 0.12 to 0.15 mg·kg^−1^ of d.w., and there were significant differences between groups I-II and II-III. Among all analysed elements, the coefficient of variation was lowest for Cd.

In order to demonstrate the relationship between the concentrations of toxic and essential trace elements in the horse feed, Table 4 presents correlation coefficients for all study groups. Analysis revealed a significant positive correlation for Cu-Pb (r_xy_ = 0.723) and Zn-Cd (r_xy_ = 0.714) at *p* ≤ 0.01.

Additionally, Table 5, Table 6, Table 7 and Table 8 present interactions between the concentrations of the same toxic and essential trace elements in different samples of hair vs. oat. In the case of Zn and Cu, a highly significant negative interaction was obtained in all groups (r_xy_ = −0.633; r_xy_ = −0.827; r_xy_ = −0.785; r_xy_ = −0.815, respectively). Furthermore, as Pb in oats increased, the Cu content in horse hair decreased, which was statistically confirmed in group I (r_xy_ = −0.652), II (r_xy_ = −0.604) and IV (r_xy_ = −0.615). Similar correlations were recorded for Cd and Cu in research groups II-IV, where successive coefficients were at the level of r_xy_ = −0.690; r_xy_ = −0.657 and r_xy_ = −0.642, respectively. The analysis also revealed significant positive interactions between the concentrations of Cu in oats and horse hair in all study groups, (namely, one by one: r_xy_ = 0.612; r_xy_ = 0.653; r_xy_ = 0.669 and r_xy_ = 0.695). In addition, statistically significant negative interactions between Zn in oats and Pb in horse hair were obtained in the I and III research groups (r_xy_ = −0.691 and r_xy_ = −0.619, respectively).

The basic chemical composition of oats obtained from each stable where horses were kept was analysed to assess the nutritional value of the equine feed. Detailed parameters are presented in Table 9. All measured values were consistent with standards and mean values for the basic components of oat grains. No significant differences in the chemical composition of oats were found between groups.

## 4. Discussion

Any anomalies in the concentration of trace elements in hair and other tissues may be associated with an increased risk of various pathologies and dysfunctions or indicate an inflammatory process or disease [12,13,14,20]. Differences in the concentrations of trace elements may be directly influenced by many factors, including age, sex of animals, diet, as well as individual physiological differences and the status of the habitat and the associated exposure to ubiquitous xenobiotics [10,12,13,21]. In hair, compared to other analytical materials, toxins can be detected a long time after exposure, which enables their identification and preventive actions to limit exposure [10,11,12,21,22]. Our study revealed differences in the content of essential and toxic trace elements in horse hair between the study groups (Table 1). Study groups mainly differed in terms of the concentration of the analysed metals in oats, which is the basic component of the horse diet (Table 3), and types of physical activity related to the way horses were used (see description of the study groups). A study by Farmer and Farmer indicated a clear relationship between the concentrations of Zn, Cd and Pb in the feed and the concentrations of these elements in the hair, kidneys and liver of cattle, sheep and horses. The concentrations of Pb in horse hair reported by these authors [23] were almost 6-fold higher than those measured in our study. The concentration of Zn was similar, whereas the concentration of Cd, due to the high concentration of habitat pollution, exceeded 4 mg · kg^−1^, which was significantly higher than in our study. Heavy metal concentrations in the hair of dairy cows reported by Miroshnikov et al. were similar to those measured in our study [24]. Ghorbani et al. [11] also confirmed, among other things, a significant relationship between the concentrations of Cu and Zn in feed and their concentrations in horse hair. Our research also demonstrated a significant interaction between the Cu content in feed and the concentration of this element in horse hair (r_xy_ = 0.695; *p* ≤ 0.05). The comparison of oats and hair samples for Zn-Cu concentrations revealed a significant negative interaction between these elements (Table 5, Table 6, Table 7 and Table 8), which means that Zn content in horse hair decreased with the increased dietary intake of Cu. The analysis of horse hair also showed the same negative and significant interaction between Zn and Cu (Table 2). This can be explained by the metabolic antagonism between these elements that compete with each other during their absorption from the gastrointestinal tract. The presence of copper influences the concentration of zinc in the body and its metabolism [2,3,25,26]. The same negative and significant correlation was reported from studies on the hair of red deer [3] and roe deer [2]. On the other hand, the high content of zinc in the diet reduces the accumulation of copper in the body. Zinc regulates the synthesis of metallothionein. A high dietary intake of zinc, and thus its higher concentration in the body, is associated with the formation of metallothionein–copper complexes of limited potential for absorption, which leads to faster copper elimination [9,25]. Our study also revealed a negative interaction between the content of zinc in oats and the content of copper in hair, but the detected differences were not significant (Table 5, Table 6, Table 7 and Table 8). Ghorbani et al. reported a 30% lower zinc content, 50% lower copper content and 50% higher lead content in horse hair compared to our findings, and these results, with the exception of lead, were also directly influenced by the diet [11]. Our analyses confirmed a significant interaction between the same elements in the feed-hair system for Cu-Cu. Thus, the content of Cu in the feed influenced the content of Cu in the hair (Table 5, Table 6, Table 7 and Table 8). For the other elements, the direction of interaction was identical but not statistically significant. This confirms the assumptions that the concentrations of trace elements in the diet have a significant impact on their concentration in analysed hair and other biological materials [11,23,27]. Apart from these relationships, the most important aspect is the assessment of heavy metal content in the feed. This is particularly relevant in cases of high exposure to heavy metals via the gastrointestinal tract. The content of heavy metals in oat samples did not exceed the maximum tolerable concentration established by the National Research Council (NRC) [28], and the basic chemical composition of oats (Table 9) did not differ from the widely accepted standards [19]. The quality of the analysed oats was satisfactory, considering the recommendations on feed for horses, and was suitable for dietary use. The horses in the presented study were fed according to specific recommendations and standards and were provided with all the necessary nutrients. As a result, the animals were healthy and in good shape, as confirmed by the veterinarian. This study focused mainly on investigating the relationship between the concentrations of elements in individual analytical materials and on their interactions. The concentrations of lead and cadmium in the hair measured in our study were within the reference values for healthy horses [20]. The concentrations of zinc and copper measured in our study were 50% and 30% higher, respectively, compared to the concentrations reported by Asano et al. [20]. With reference to other studies by Asano et al. [29,30], the concentrations of Zn, Pb and Cu were comparable to our findings.

Other important interactions confirmed in the presented study concerned the content of lead and cadmium in oats and the content of copper in horse hair. Increased concentrations of lead and cadmium in oats were associated with decreased concentrations of copper in horse hair (Table 5, Table 6, Table 7 and Table 8). A significant correlation for Cd-Cu (r_xy_ = −0.676) was also found in hair samples (Table 2). A similar trend was reported by Cai et al. (2009) in bovine liver, where increased concentrations of lead and cadmium were associated with decreased concentrations of copper. Moreover, overexposure to these toxic metals in the cited studies resulted from environmental pollution and their high concentrations in plants ingested by animals [4]. Copper competes with cadmium and lead for absorption from the gut and distribution in tissues. In addition, cadmium has the strongest impact on the metabolism of copper, which may explain the decrease in tissue copper concentrations in animals at high exposure to cadmium [4,31].

In general, differences in the concentrations of toxic and essential elements in different analytical materials are directly related to the level of exposure of animals to xenobiotics, which can enter the body via the gastrointestinal and respiratory tracts [2,6,8]. In our study, the differences between the research groups could have been influenced not only by feed, but also by different types of physical activity directly related to the use of horses. The increase in the level of sport activity of horses is associated with changes in the concentration of some elements [15]. A study by Kalashnikov et al. confirmed the decrease in the concentrations of toxic metals, including cadmium and lead, in the hair of horses who were more active [15]. In our study, lower concentrations of these heavy metals were also found in the hair of sports horses compared to horses used less intensively (Table 1). However, this issue requires further research to better understand such relationships.

Taking into account the fact that animals from all study groups lived in the same region characterised by the same level of environmental pollution, which, especially in the case of toxic metals, i.e., lead and cadmium, was not exceeded for the entire area of the Kuyavian-Pomeranian province [17], the environmental exposure of each group of horses can be regarded as identical. Despite the fact that in our research this factor was of little importance, the state of the natural environment certainly has a considerable impact on the accumulation of heavy metals in tissues, which has been confirmed by many authors [2,4,23,24].

Our study did not reveal any significant sex-related differences in the concentrations of analysed elements, and therefore the analyses were performed for female and male horses together in individual study groups. Asano et al. [20] also reported no sex-related differences in the concentrations of essential and toxic trace elements. On the other hand, other studies revealed that sex may influence the concentration of these elements in analytical matrices, including hair [2,11].

When breeding animals, it is particularly important to monitor the chemical composition of their feed, both cereal grains and hay. Complementary knowledge in this field is essential for full control over exposure and the health of horses. Periodic health checks of animals, including blood tests in combination with an optimised diet, ensure optimal outcomes in horse breeding, regardless of the way these animals are used.

## 5. Conclusions

Horse hair used as an analytical matrix for the monitoring of heavy metal concentrations can be a source of important information for the assessment of exposure and the health of these valuable animals. Findings from our study confirmed the suitability of horse hair analysis for monitoring of animal exposure and health. Low concentrations of toxic metals, including lead and cadmium, indicated that horses were not overexposed to these elements. The concentrations of selected trace elements in oats had a significant effect on their content in the hair, which was confirmed by numerous interactions. Higher concentrations of essential and toxic trace elements in the hair in individual study groups may indicate higher exposure to these elements or higher potential for accumulation. Differences in the types of physical activity between groups of horses could influence the biotransformation of these trace metals and thus cause their different concentrations in the analysed material. Horses from different study groups lived in the same region, which was characterised by similar levels of environmental pollution, so this factor was not considered as a variable in statistical analysis. Despite the fact that the horses were used for different purposes and received feed from different sources, none of the study groups was overexposed to heavy metals, which was confirmed by analytical findings. Concentrations of heavy metals measured in the presented experimental study did not exceed acceptable concentrations established for these elements.

## Figures and Tables

**Table 1:** Content of selected essential and toxic trace elements in the hair of horses [mg·kg^−1^ of d.w.].

Trace Elements		Research Groups			
		I	II	III	IV
		*n* = 8	*n* = 9	*n* = 9	*n* = 10
Zn [mg·kg^−1^]	$\bar{x}$	156.45 ^ab^	153.56 ^a^	165.62 ^ab^	185.79 ^b^
	Me	157.27	155.86	164.22	178.61
	SE	±8.201	±6.478	±7.907	±8.797
	CV	20.967	17.898	20.254	21.176
Cu [mg·kg^−1^]	$\bar{x}$	11.997 ^A^	6.104 ^B^	8.330 ^B,C^	9.394 ^A,C^
	Me	11.205	6.563	8.347	9.392
	SE	±1.240	±0.486	±0.727	±0.527
	CV	41.352	33.78	37.032	25.11
Pb [mg·kg^−1^]	$\bar{x}$	0.579 ^a^	0.713 ^a^	0.813 ^a^	0.578 ^a^
	Me	0.305	0.536	0.728	0.366
	SE	±0.168	±0.137	±0.128	±0.105
	CV	76.24	81.63	70.63	76.99
Cd [mg·kg^−1^]	$\bar{x}$	0.013 ^a,b^	0.012 ^a^	0.015 ^b^	0.011 ^a^
	Me	0.013	0.011	0.014	0.011
	SE	±0.0004	±0.0001	±0.0009	±0.000
	CV	13.302	4.268	25.777	14.390

^a, b^—values marked with different letters in the same row differ significantly (*p* ≤ 0.05); ^A,B,C^—values marked with different letters in the same row differ significantly (*p* ≤ 0.01), Me-Median (Q_2_); SE—standard error; CV (%)—coefficient of variation; *n*—number of animals; I, II, III, IV—stable number.

**Table 2:** Correlation coefficients (r_xy_) for the concentrations of selected essential and toxic trace elements in the hair of horses.

Trace Elements	Cu	Pb	Cd
Zn	−0.539 *	0.034	−0.001
Cu		0.261	−0.676 *
Pb			−0.077

* Correlation coefficients were significant at *p* ≤ 0.05.

**Table 3:** The content of selected essential and toxic trace elements in the oats [mg·kg^−1^ of d.w.].

Trace Elements		Research Groups			
		I	II	III	IV
		*n* = 8	*n* = 8	*n* = 8	*n* = 8
Zn [mg·kg^−1^]	$\bar{x}$	75.677 ^a^	87.84 ^a^	66.295 ^a^	58.063 ^a^
	Me	55.952	91.487	65.351	53.364
	SE	±17.062	±16.317	±7.011	±7.831
	CV	63.770	52.540	29.913	38.149
Cu [mg·kg^−1^]	$\bar{x}$	19.357 ^A^	13.315 ^B^	12.114 ^B^	11.846 ^B^
	Me	20.69	12.690	12.335	11.820
	SE	±0.919	±3.193	±0.581	±1.051
	CV	19.516	46.651	13.567	25.091
Pb [mg·kg^−1^]	$\bar{x}$	0.495 ^a^	0.636 ^a^	0.499 ^a^	0.582 ^a^
	Me	0.440	0.575	0.365	0.589
	SE	±0.118	±0.152	±0.099	±0.074
	CV	67.523	67.538	55.853	36.078
Cd [mg·kg^−1^]	$\bar{x}$	0.123 ^a^	0.152 ^b^	0.117 ^a^	0.131 ^a,b^
	Me	0.122	0.512	0.116	0.129
	SE	±0.004	±0.013	±0.002	±0.002
	CV	8.22	23.380	4.819	3.532

^a, b^—values marked with different letters in the same row differ significantly (*p* ≤ 0.05); ^A, B^—values marked with different letters in the same row differ significantly (*p* ≤ 0.01), Me—Median (Q_2_); SE—standard error; CV (%)—coefficient of variation; *n*—number of samples; I, II, III, IV—stable number.

**Table 4:** Correlation coefficients (r_xy_) for the concentrations of selected essential and toxic trace elements in the oats.

Trace Elements	Cu	Pb	Cd
Zn	−0.404	−0.071	0.714 **
Cu		0.723 **	−0.419
Pb			0.095

** Correlation coefficients were significant at *p* ≤ 0.01.

**Table 5:** Correlations between the concentrations of the same elements in different samples of hair vs. oat-interaction coefficients (Research group I).

Trace Elements	Zn (Oat)	Cu (Oat)	Pb (Oat)	Cd (Oat)
Zn (hair)	0.357	−0.633 *	−0.047	−0.525
Cu (hair)	−0.238	0.612 *	−0.652 *	−0.419
Pb (hair)	−0.691 *	0.383	0.346	−0.166
Cd (hair)	0.235	−0.341	0.414	0.404

Interaction coefficients were significant at *p* ≤ 0.05 *.

**Table 6:** Correlations between the concentrations of the same elements in different samples of hair vs. oat-interaction coefficients (Research group II).

Trace Elements	Zn (Oat)	Cu (Oat)	Pb (Oat)	Cd (Oat)
Zn (hair)	0.194	−0.827 **	−0.378	−0.166
Cu (hair)	−0.357	0.653 *	−0.604 *	−0.690 *
Pb (hair)	−0.381	0.386	0.261	−0.476
Cd (hair)	0.366	−0.011	0.296	0.309

Interaction coefficients were significant at *p* ≤ 0.05 * and *p* ≤ 0.01 **.

**Table 7:** Correlations between the concentrations of the same elements in different samples of hair vs. oat-interaction coefficients (Research group III).

Trace Elements	Zn (Oat)	Cu (Oat)	Pb (Oat)	Cd (Oat)
Zn (hair)	0.162	−0.785 **	−0.219	−0.537
Cu (hair)	−0.501	0.669 *	−0.412	−0.657 *
Pb (hair)	−0.619 *	0.476	0.243	−0.264
Cd (hair)	−0.107	−0.327	0.268	0.071

Interaction coefficients were significant at *p* ≤ 0.05 * and *p* ≤ 0.01 **.

**Table 8:** Correlations between the concentrations of the same elements in different samples of hair vs. oat-interaction coefficients (Research group IV).

Trace Elements	Zn (Oat)	Cu (Oat)	Pb (Oat)	Cd (Oat)
Zn (hair)	0.042	−0.815 **	−0.451	−0.592
Cu (hair)	−0.047	0.695 *	−0.615 *	−0.642 *
Pb (hair)	−0.452	0.368	0.047	0.011
Cd (hair)	−0,311	−0.190	0.202	0.252

Interaction coefficients were significant at *p* ≤ 0.05 * and *p* ≤ 0.01 **.

**Table 9:** Basic chemical composition of oats [%].

Parameters		Research Groups			
		I	II	III	IV
		*n* = 8	*n* = 8	*n* = 8	*n* = 8
Dry weight [%]	$\bar{x}$	89.89 ^a^	89.64 ^a^	89.87 ^a^	89.91 ^a^
	SE	±0.021	±0.035	±0.045	±0.031
	Standard	88%			
Protein [%]	$\bar{x}$	14.31 ^a^	10.25 ^a^	11.84 ^a^	10.56 ^a^
	SE	±0.421	±0.324	±0.214	±0.124
	Standard	10.4%			
Fat [%]	$\bar{x}$	4.09 ^a^	4.62 ^a^	4.41 ^a^	4.51 ^a^
	SE	±0.124	±0.041	±0.087	±0.045
	Standard	4.1%			
Fiber [%]	$\bar{x}$	8.08 ^a^	8.23 ^a^	7.79 ^a^	8.06 ^a^
	SE	±0.245	±0.045	±0.478	±0.224
	Standard	8.9%			
Ash [%]	$\bar{x}$	4.08 ^a^	3.52 ^a^	3.44 ^a^	3.74 ^a^
	SE	±0.025	±0.072	±0.014	±0.042
	Standard	3.2%			
Starch [%]	$\bar{x}$	46.36 ^a^	47.72 ^a^	48.80 ^a^	51.23 ^a^
	SE	±0.756	±0.951	±0.545	±0.654
	Standard	45%			

^a^—values marked with different letters in the same row differ significantly (*p* ≤ 0.05); SE—standard error; Standard—recommendations for selected chemical components [19].

## Data Availability

The data presented in this study are available on request from the corresponding author.
